# Aortic Arch Baroreceptor Stimulation in an Experimental Goat Model: A Novel Method to Lower Blood Pressure

**DOI:** 10.3389/fcvm.2018.00193

**Published:** 2019-01-15

**Authors:** Jacobus F. Benson, Johan P. Schoeman, Frans J. Venter, James A. Ker, Gareth E. Zeiler, Lynette Bester, Janet van Niekerk, Gregory R. Tintinger

**Affiliations:** ^1^Faculty of Health Sciences, University of Pretoria, Pretoria, South Africa; ^2^Faculty of Veterinary Science, University of Pretoria, Pretoria, South Africa; ^3^College of Public Health, Medical and Veterinary Sciences, James Cook University, Townsville, QLD, Australia; ^4^Department of Statistics, University of Pretoria, Pretoria, South Africa

**Keywords:** aortic baroreceptor stimulation, autonomic nervous system, carotid baroreceptors, hypertension, systolic blood pressure

## Abstract

The effect of aortic baroreceptor stimulation on blood pressure manipulation was assessed using the goat species *Capra aegagrus hircus*. The aim of this study was to manipulate blood pressure with future intention to treat high blood pressure in humans. The ages of the animals ranged from 6 months to 2 years. A standard anesthesia protocol was used. A lateral thoracotomy was performed to gain access to the aortic arch. Data was collected with the Vigileo system. Pre stimulation blood pressure was compared with maximum post stimulation blood pressure values. Results were analyzed with the Wilcoxon signed rank test. In the study 38 animals were enrolled. Baroreceptor stimulation was performed for each animal using 3 different electrodes each of which emits an electrical impulse. In the pilot phase of the study, the median baseline blood pressure prior to stimulation of the baroreceptors was 110.8 mmHg. After stimulation the median blood pressure decreased to 88 mmHg. The average decrease in blood pressure was 22.8 mmHg. This decrease of blood pressure after stimulation of the baroreceptors is statistically significant (*p* < 0.0001) and the proof of concept was shown. During the extended phase all three probes had a significant effect on blood pressure lowering (*p* < 0.0001). The study confirmed that aortic baroreceptor stimulation has an effect on blood pressure lowering. This is a novel field of blood pressure manipulation. The hemodynamic effects of long-term aortic baroreceptor stimulation are unknown. Further investigations need to be done to determine whether a similar effect can be induced in different species such as primates and humans.

## Introduction

Arterial hypertension in humans is the most important risk factor for cardiovascular disease, premature death, and disability globally (1). Despite the effect of potent pharmacological drugs in treating blood pressure, a substantial number of patients do not achieve goal blood pressure values. The cardiovascular diseases include coronary heart disease, stroke, heart failure, and arrhythmias (such as atrial fibrillation) (2–4). Various drug trials and life-style change studies showed significant reductions in cardiovascular events, yet globally hypertension remains poorly controlled, with less than a third of patients achieving target blood pressure (1, 5). Data from the National Health and Nutrition Examination Survey (NHANES 2013–2014) also demonstrated an increased incidence of hypertension (6). These observations led to the search for alternative measures for blood pressure control such as surgical intervention by implanting an aortic baroreceptor stimulation device.

In 2015 it was estimated that the global prevalence of hypertension is 1.13 billion worldwide and that 150 million patients in Europe suffer from hypertension. The estimated increase of people with hypertension by 2025 is to be a number of 1.5 billion (7). The financial burden on the health care system in direct and indirect cost in the United States of America in 2008 was $50 billion and impacted more than 76 million Americans (8).

An increased activity of the sympathetic nervous system, in part regulated by afferent input arising from arterial and cardiopulmonary baroreceptors, has been demonstrated to play a critical role in developing and sustaining hypertension (9). Activation of these receptors causes inhibition of the sympathetic system and potentially can reduce blood pressure.

Recently, electrical stimulation of the carotid sinus showed encouraging results in lowering blood pressure and reducing sympathetic drive. This was achieved both in short-term and long-term period of over 5 years and may have the potential to become a novel therapy for hypertension (10).

A variety of causes can lead to hyper-activity of the sympathetic nervous system - one such cause is inactive baroreceptors. Low-pressure baroreceptors have an effect on renal-endocrine and circulatory systems effecting retention of salt and water. In addition, the neuro hormonal system resets the baroreceptor set point over time and this baroreceptor dysfunction consequently leads to an increase in blood pressure (11).

Baroreceptors are located in blood vessels of all vertebrate animals. The sensory nerve endings of arterial baroreceptors are simple sprayed endings that lie in the adventitia of an artery. These baroreceptors function as a type of mechanoreceptor sensory system. Increase in the arterial pressure and thus stretching of the artery, results in action potentials triggered in these baroreceptor endings. Baroreceptors provoke action potentials with each heartbeat. These action potentials cause neuron stimulation which is conducted to the central nervous system mainly via the autonomic nervous system (8, 12).

This neuron stimulation modulates the heart rate, inotropic activity, thus also cardiac output as well as vascular tone in the form of peripheral resistance (8).

The release of hormones, such as adrenaline, that targets cardiac function, can affect these baroreceptors instantly via sympathetic pathways. Baroreceptors respond immediately to maintain a stable blood pressure. Mean arterial pressure (MAP) and pulse pressure influence nerve firing so that the autonomic nerve system maintains near to the normal range of MAP. Physiological changes in the set point of blood pressure control occur in physical activity, hypertension, and heart failure (9, 12–14). Adjustment over time occurs in this system, which alters the sensitivity of these receptors. Changes in the set point explain why elevated blood pressure is maintained during exercise and in patients with chronic hypertension (15).

Previous investigators have evaluated the effect of carotid baroreceptor stimulation on hypertension (16). New areas investigated in baroreceptor activity included hypertension treatment (carotid stimulation), sleep apnoea syndrome, and even diabetes control. This needs to be investigated further (10, 17). During these investigations it was found that diabetes control and sleep apnoea improved.

Carotid baroreceptor stimulation has already been evaluated in a double blind randomized placebo controlled Rheos Pivotal Trial in 2012 by Bakris. These results showed significant benefit for endpoints of sustained efficacy (18).

The Barostim neo trial showed that a second-generation, minimally invasive system for baroreflex activation therapy (BAT) led to a significant reduction in SBP. It was then stated that this therapy is the only treatment that demonstrated long-term safety and efficacy in a large-scale double blind, randomized control trial for the treatment of resistant hypertension (8, 10). In 2012 Bloch et al. showed that there is controversy regarding the procedural safety of the device and the implantation procedure (19).

BAT therapy is intensely evaluated in resistant hypertension with excellent results in lowering blood pressure. The Reos Biostim system was developed for the treatment of resistant hypertension. Further studies of BAT investigated the long-term effect of this modality on heart failure with reduced ejection fraction (20–22). A further study on the effects of chronic carotid baroreceptor activation on arterial stiffness in severe heart failure was published in 2016 (23).

The rationale for using aortic arch baroreceptor stimulation in this study is based on a study that was done on the sensitivity between aortic baroreceptors and carotid baroreceptors indicating that the aortic depressor neurons possessed a higher percentage of mechano-sensitive neurons (24). Therefore, we postulate that aortic baroreceptor stimulation may be more effective in lowering blood pressure.

Stimulation of the aortic arch baroreceptors has not been previously investigated. This research study aimed to investigate a novel mechanism in blood pressure manipulation through aortic arch baroreceptor stimulation with the hypothesis that stimulation of aortic arch baroreceptors will cause a significant reduction in systolic blood pressure (SBP).

## Materials and Methods

### Study Design

The study was designed as a proof of concept. It was approved by the University of Pretoria Biomedical Research Council (Project no H007/14). Initially, during the pilot study, a total of 21 animals were subjected to aortic baroreceptor stimulation and the blood pressure response was measured. During the extension of the study the efficacy of blood pressure lowering was evaluated with three different probes in 38 experimental animals of the goat species *Capra aegagrus hircus*. This species was chosen due to the convenient size, especially of the aortic arch and consequently the ease to identify the sites of the aortic baroreceptors. Both sexes were included in this study. The age of the animals varied from 6 months to 2 years.

### Anesthetic Protocol

All research animals received general anesthesia. The anesthetic induction and procedure included a single bolus of Alfaxalone (5 mg/kg) combined with intramuscular Midazolam (0.5 mg/Kg) via a jab stick. This was followed by intubation through which Isoflurane and oxygen were delivered. Maintenance fluid (Lactated Ringers solution) was administered via an auricular vein at 10ml/kg with a Braun infusion pump.

The femoral artery catheter was connected to the Vigileo system that measured blood pressure intra-arterially. After five stable blood pressure measurements at 5 min intervals, baroreceptor stimulation was initiated. The duration of stimulation varied between animals and was continued until the lowest SBP was observed. The duration of stimulation ranged from 15 to 45 min. Stimulation was applied until maximum SBP was achieved. The protocol did not include a recovery phase.

Subsequent measurements were taken at 5 min intervals until the lowest blood pressure value was recorded. All animals were euthanized after the intervention according to protocol requirements.

### Surgical Procedures

This procedure consisted of left lateral intercostal thoracotomy incision at the level of the 3rd and 4th ribs. A bolus of Buprenorphine (0.02 mg/kg IV) was administered prior to the surgical procedure to minimize the effect of pain on SBP. Subsequently, the aortic arch was dissected down to the adventitia layer. The probe was then placed on the outer surface of the aortic arch at the level of bifocation of the aorta into dorsal and ventral vessels. This is the usual anatomical location of the aortic baroreceptor in goats. The position of the probe was confirmed by direct visual inspection.

### Adverse Events

Adverse events that were monitored included major bleeding during the surgical exposition and during the stimulation phase. One major bleeding occurred due to aortic perforation. Blood pressure drop during the initiation phase of anesthesia of the animals was monitored as was provided by the anesthesia protocol.

### Probe Design

The Octad probe is designed as an eight contact probe, bended to fit over the aorta. The eight contacts provide optimum contact area to cover the baroreceptors on the aortic wall. This consists of probe length of 44.5 mm, inter-electro spacing of 6 mm, electrode size of 2.4 x 3.8 mm and electro design of 2 rows of 4 electrodes staggered.

The Quad probe is designed with four contact areas. The probe length is 44mm with inter electrode spacing of 5 mm, electrode size of 2 × 2 mm and inline design of one row of 4 contacts.

The Pace probe is designed as a single tip stimulation probe with a tip size of 1 mm^2^.

### Stimulation Procedure

Stimulation of the aortic arch was performed using the Octad, Quad and Pace probes at a voltage and frequency of 10 mvolts and 60 Hertz, respectively.

Data was collected using the Vigileo System (Edwards Lifesciences, Irvine, California, United States) which is a hemodynamic monitoring system that allows monitoring of blood pressure and pulse rate. This system was connected to a femoral artery cannulation probe. Blood pressure response was measured via the arterial pressure wave of the femoral artery. Blood pressure measurements were recorded at 5 min intervals in order to determine the effect of aortic baroreceptor stimulation on blood pressure and pulse rate.

### Ethical Approval

These processes were done after best practice methods had been approved by the ethics committee of UPBRC (University of Pretoria Animal Ethics Committee registration with the National Health Research Ethics Council: AREC-261110-001). UPBRC facility registration number with the South African Veterinary Council: FR15/1383.

### Primary Endpoints

The primary end-point was the maximum reduction in SBP that could be achieved with baroreceptor stimulation (proof of concept). In the extension phase of the study the endpoint was to evaluate the effect of three different probe designs on SBP lowering.

### Statistical Analysis

As per trial design assumptions, sample size was calculated to adequately power endpoint results for a power of 90% at the significance level of 0.05. Sample size was determined as 17 experimental animals. Twenty one animals were included in the study. The first four animals were used to determine the technical aspects. These four animals were included in the final data analysis.

For this research the null hypothesis of no relationship between stimulation of aortic baroreceptors and a reduction in blood pressure was applied. By rejecting the null hypothesis would then conclude that there are grounds for believing that there is a relationship between stimulation of the receptors and the lowering of blood pressure. The null hypothesis was rejected if a significant blood pressure lowering, defined as a 20 mmHg decrease in blood pressure, was achieved. The control measure is defined as the blood pressure reading before stimulation of the receptors. The effect is measured by applying the relevant statistical tests as described below.

A significant reduction in SBP of 20 mmHg (due to stimulation) sample size determination was applied. A standard deviation of 23.62 mmHg is assumed (√2^*^ expected range for reduction in SBP/∂ = √2 ^*^ 100/∂). A sample size of 17 animals had 90% power to detect a change from baseline of at least 20 mmHg when the standard deviation is 23.62 mmHg and testing is two-sided at the 0.05 level of significance. During this study baseline and endpoint blood pressure results were compared.

The final results were analyzed with the Kolmogorov-Smirnov test that showed that both pre-stimulation and post-stimulation data were non-parametric. The Wilcoxin Signed-Rank Test (SAS Version 9.4) was therefore applied to achieve the final results. Statistical analysis of the study was done by the Statistical Department at the University of Pretoria.

With further analysis the Friedman (compare=pairwise) test was applied to show the differences between the three probes.

Three probes were used and allocated according to a numeric randomized design. Pre- and post- stimulation readings were collected manually for each probe.

## Results

During the pilot phase and as proof on concept 21 animals were used. The initial four animals were allocated to perfect the technique. Ultimately, the variance was so low that the data from these four animals were added to the final data as per protocol.

**Figure 1:**
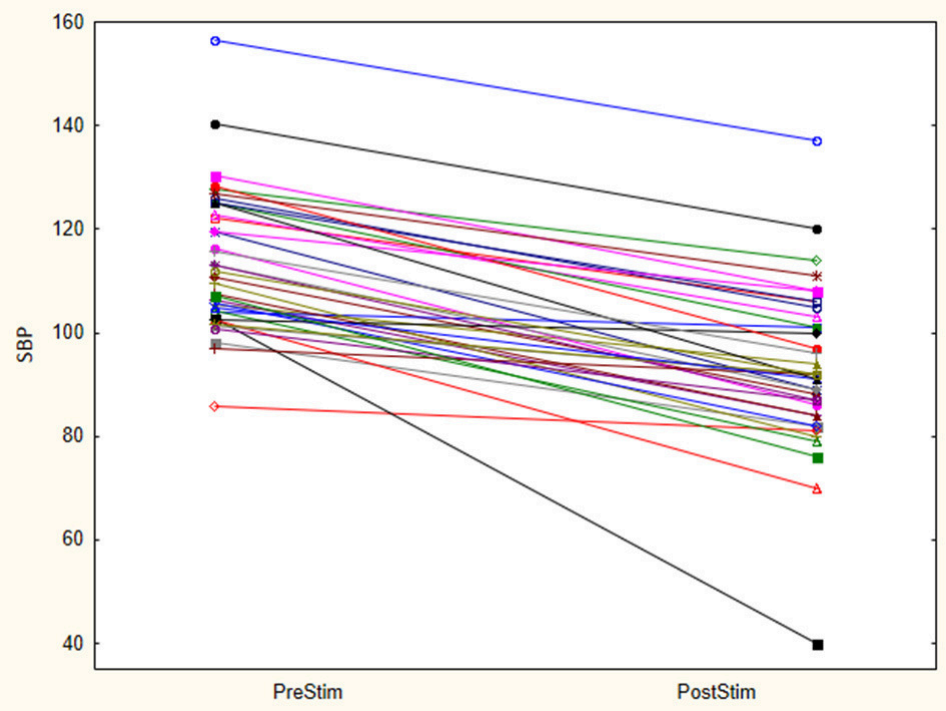
Stimulation results- Octad probe (pilot phase). Results of pre and post stimulation for each research animal are plotted in this figure (*n* = 21). These are the results of aortic arch baroreceptor stimulation with the Octad experimental probe. SBP blood pressure values are represented in mmHg.

**Figure 2:**
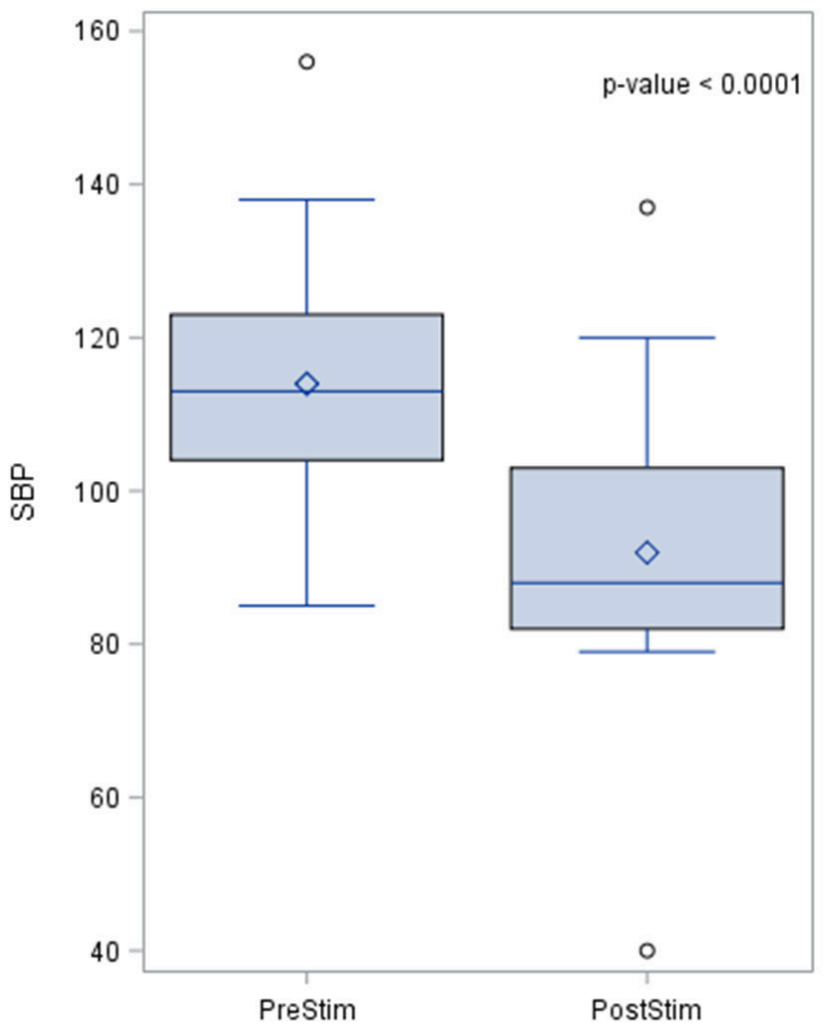
The box plot for the results of systolic blood pressure lowering in the pilot phase using the Octad probe. The results of the distribution of the median are shown in the figure, together with the *p*-value after applying the Wilcoxin rank test. Systolic blood pressure values are represented in mmHg.

The baseline SBP before stimulation had a median value of 110.8 mmHg with a standard deviation from the mean of 13.67 mmHg. After stimulation, SBP was 88 mmHg with a standard deviation of 15.93 mmHg from the mean. This showed a significant lowering of SBP with a median reduction of 22.8 mmHg. Pre- and post-stimulation values for each animal are shown in Figure 1. The box plot for the results of SBP lowering in the pilot phase is shown in Figure 2. The Octad probe was used during the pilot phase.

**Figure 3:**
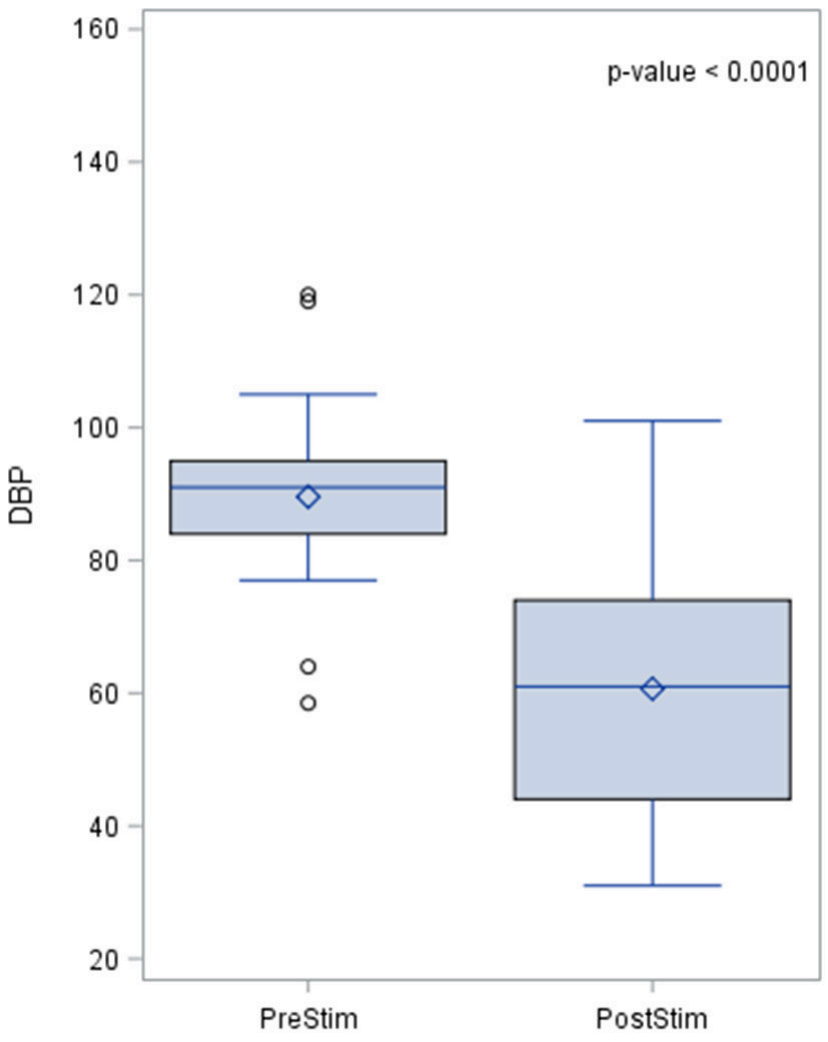
Effects of aortic baroreceptor stimulation on diastolic blood pressure. The results of the distribution of the median are shown in the figure, together with the *p*-value after applying the Wilcoxin rank test. Diastolic blood pressure values are represented in mmHg.

The effects of aortic baroreceptor stimulation on diastolic blood pressure (DBP) are shown in Figure 3. The pre-stimulation value of DBP had a median of 90.0 mmHg with a standard deviation from the mean of 15.1 mmHg. The DBP post-stimulation was had a median of 16 mmHg with a standard deviation from the mean of 17.25 mmHg.

**Figure 4:**
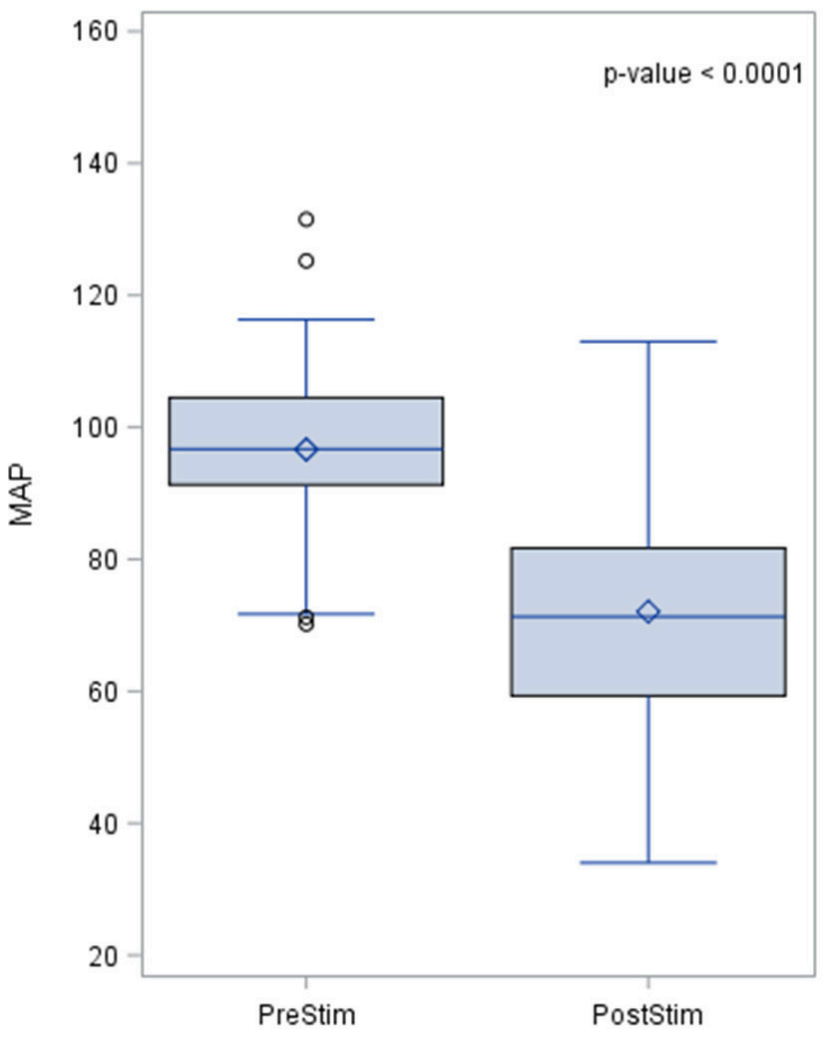
Effects of baroreceptor stimulation on mean arterial pressure. The results of the distribution of the median are shown in the figure, together with the *p*-value after applying the Wilcoxin rank test. Mean arterial pressure values are represented in mmHg.

The MAP pre- and post-stimulation is shown in Figure 4. The median value pre-stimulation was 96.7 mmHg and post-stimulation was 71.3 mmHg with a standard deviation from the mean of 14 and 15.94, respectively.

**Figure 5:**
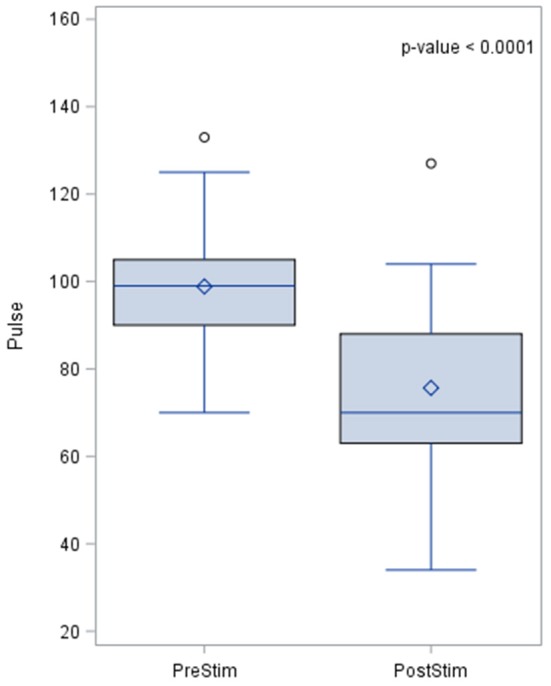
Effects of baroreceptor stimulation on pulse rate. The results of the distribution of the median are shown in the figure, together with the *p*-value after applying the Wilcoxin rank test. Pulse rate values are presented in beats per minute.

Pulse rate also changed during baroreceptor stimulation and the pre- and post-stimulation results are shown in Figure 5. The pre-stimulation value of the pulse rate was a median of 99.0 beats per minute (bpm) with a standard deviation from the mean of 14.5. The post-stimulation value of the pulse rate was a median of 70 bpm and the standard deviation of 20 bpm which indicates a lowering of pulse rate after stimulation.

In the extension phase of the study three probes (Octad, Quad and Pace) were used to evaluate whether a different probe design would have a similar effect on blood pressure lowering.

**Figure 6:**
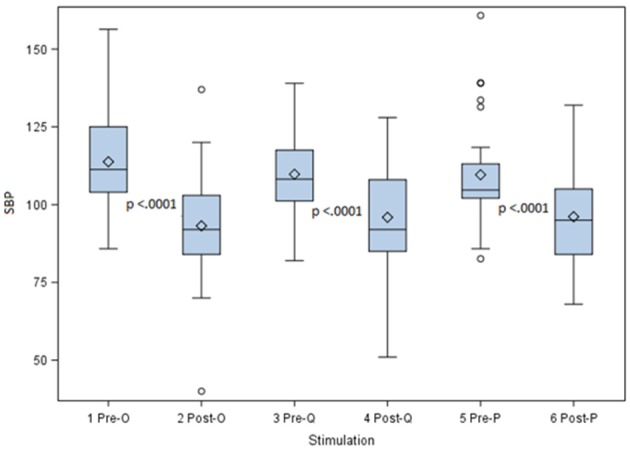
Pre- and Post- stimulating SBP for three different stimulation probes. This figure shows the systolic blood pressure lowering for the Octad, Quad, and Pace probes. With all three stimulation probes a highly significant lowering in blood pressure was achieved with a *p*-value = 0.0001.

Data for the different probes were non-parametric with the skewness coefficients of 0.8 (probe O) 0.18 (probe Q) and 4.5 (probe P), respectively. The distribution of the pre- and post- stimulating SBP for the 3 probes is presented in Figure 6. For the Octad probe the median blood pressure before stimulation was 110.8 mmHg (standard deviation of 13.62) and the median blood pressure lowering after stimulation was 22.8 mmHg (standard deviation = 10.9).

For the Quad probe the median blood pressure before stimulation was 108.2 mmHg (standard deviation = 13.14) and after stimulation the blood pressure lowering had a median value of 12.2 mmHg (standard deviation = 11.5).

For the Pace probe the pre-stimulation median blood pressure was 104.7 mmHg (standard deviation = 37.1). After stimulation the median lowering value was 12.4 mmHg (standard deviation 15.48).

With all three stimulation probes, despite the difference in design, a highly significant lowering in blood pressure was achieved with a *p* = 0.0001 according to the Wilcoxin Signed-Rank test. The null hypothesis was thus rejected and aortic baroreceptor stimulation is associated with lowering in blood pressure.

**Figure 7:**
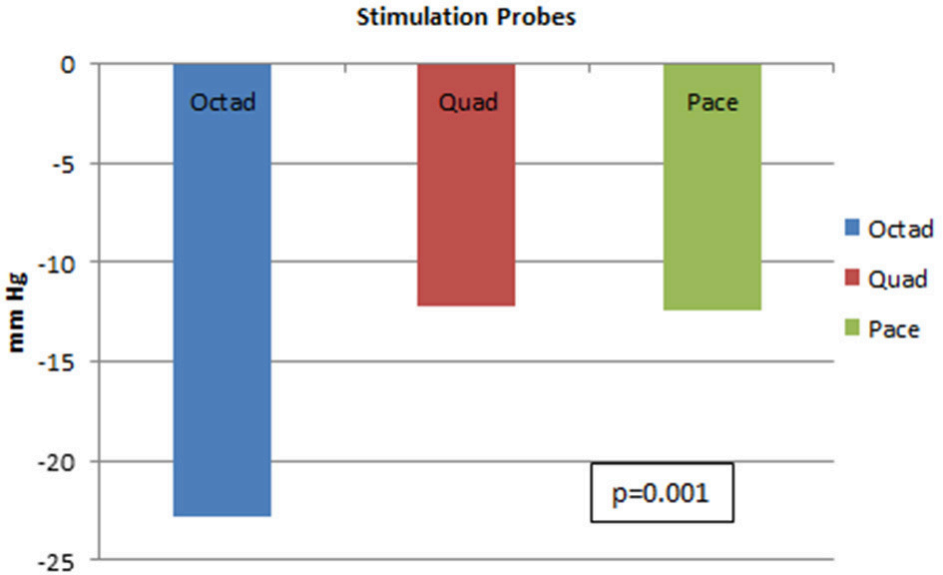
Systolic blood pressure lowering for three different stimulation probes. This figure shows the systolic blood pressure lowering for the Octad, Quad, and Pace probes. There is superiority of the Octad probe in lowering blood pressure (*p*-value = 0.001).

With further analysis on the differences between the three probes, it was proved that the Octad probe was most effective when compared to the other two probes in lowering SBP with *p*-value of 0.001. This result is shown in Figure 7.

## Discussion

The findings of the current study suggest that stimulation of aortic baroreceptors in an animal model may represent a viable method to decrease arterial pressure. Stimulation of aortic baroreceptors induced a significant decrease in systolic, diastolic, and mean arterial pressure in this animal model.

Hypertension in humans is an important cause of morbidity and mortality worldwide and significant resources are utilized annually to treat hypertension, (1). However, compliance with pharmacological therapy is relatively poor and few patients achieve target blood pressure (25, 26). Furthermore, the side effects of medication contribute to poor compliance and some patients with resistant hypertension require multiple agents (25, 27–29).

Aortic baroreceptor stimulation caused a significant decrease in SBP in all animals tested. Importantly, the median decrement in SBP observed in this study was highly significant with a magnitude of 22.8 mmHg and was sustained for the duration of the procedure with one serious adverse effect only in the form of aortic perforation with the Pace-probe. The tip design of the Pace probe might have caused this complication. General anesthesia and appropriate analgesics were administered to the animals to minimize sympathetic stimulation during the procedure. The blood pressure response to baroreceptor stimulation was carefully monitored to ensure the accuracy of all measurements taken.

All three probes caused significant reduction in SBP with median decrements of 22.8, 12.2, and 12.4 mmHg for the Octad, Quad, and Pace probes, respectively. The Octad probe was most effective when compared to the other two probes. This may be attributed to an increased surface area of the Octad probe which has 8 stimulatory electrodes compared to the Quad and Pace probes with 4 electrodes and 1 electrode, respectively. These findings may be important in determining the design and clinical efficacy of aortic baroreceptor stimulation probes for use in human subjects.

The baroreceptor stimulation device physiologically activates the efferent reflex reducing sympathetic activity and increase parasympathetic out-flow. This leads to lowering of SBP, DBP, MAP and pulse rate as was proven in this study.

Although the carotid stimulation has been used previously for the treatment of resistant hypertension and heart failure there is no data available comparing carotid and aorta stimulation techniques (20–22).

The current study did not evaluate the effect of general anesthesia on baroreceptor stimulation. In addition, significant autonomic sympathetic activity may transiently overcome the effects of aortic baroreceptor stimulation and the animals used in this study were healthy and normotensive. A similar study using conscious animals and animals with hypertension will be required to determine whether or not the response to aortic baroreceptor stimulation is comparable to that observed in normotensive, anesthetized animals.

Limitations of this study are firstly that anesthetized and normotensive animals were used. Future experiments in active and hypertensive animals need to be done to prove the same outcome/results. Secondly the hemodynamic effect of long-term stimulation of aortic baroreceptor is not investigated. Future studies in this regard are necessary to establish if the same blood pressure lowering can be achieved.

A less invasive approach via mediastinoscopy is planned in a vertebrate animal such as primates, is planned. The effect of aortic baroreceptor stimulation on blood pressure in humans is still unknown. This could only be investigated further if sufficient data in animal models regarding safety and efficacy can be confirmed.

Future application of this research might lead to an alternative method to treat blood pressure in the resistant hypertensive population where it is known that numerous drugs are ineffective to reduce blood pressure to target values. In addition hypertension treatment during pregnancy limits the drugs that are available to treat blood pressure optimally. This non-pharmacological method might provide an alternative way to treat hypertension in future.

In conclusion, stimulation of aortic baroreceptors caused a significant decrease in SBP, DBP MAP and pulse rate and may represent a novel and alternative modality to treat systemic blood pressure in human subjects with hypertension provided these results in an animal model are repeatable in human trials.

The potential utility of this form of therapy may include subgroups of patients with resistant hypertension or those who experience significant side effects from pharmacological therapy. A new system for aortic arch baroreceptor stimulation that involves an implant procedure is currently under research for blood pressure lowering.

## Author Contributions

The article was written by JB and revised by JK, GT, and JS. GT and JS acted as promotors for this Ph.D. manuscript. The study was designed by JB and the experiments were performed by JB, FV, GZ, and LB. Statistical analysis was performed by JvN. Research funds were offered by JB.

### Conflict of Interest Statement

The authors declare that the research was conducted in the absence of any commercial or financial relationships that could be construed as a potential conflict of interest.
